# Treatment of Generalized Anxiety Disorder with Gabapentin

**DOI:** 10.1155/2017/6045017

**Published:** 2017-12-14

**Authors:** Matej Markota, Robert J. Morgan

**Affiliations:** Department of Psychiatry and Psychology, Mayo Clinic, 200 First Street SW, Rochester, MN 55905, USA

## Abstract

Gabapentin is frequently used in the treatment of anxiety disorders. However, there are no randomized controlled trials on the effectiveness of this medication in generalized anxiety disorder (GAD), and there are only a few case reports. We present a case of a 59-year-old female with a psychiatric history of GAD. The patient discontinued benzodiazepines after more than 7 years of daily treatment which led to rebound anxiety, benzodiazepine withdrawal symptoms, and suicidal ideation. She was psychiatrically hospitalized and started on gabapentin. Over the next 10 months of outpatient follow-up, she attempted to taper off gabapentin due to personal preference to limit medications. During this time, we observed a clear dose-response pattern of gabapentin on GAD symptoms. In the absence of controlled studies, these findings may offer important information about the effectiveness of gabapentin in GAD.

## 1. Introduction

While gabapentin is increasingly being used to treat generalized anxiety disorder (GAD), little is known about its effectiveness on GAD symptoms. The patient presented here has a relatively straightforward psychiatric history, with GAD playing a prominent role. Her repeated attempts to discontinue gabapentin offer a rare opportunity to observe its effect on her symptoms at different doses. A clear pattern of remission or mild anxiety on total daily doses of gabapentin ≥ 900 mg/day and severe anxiety at doses < 600 mg/day was observed. In the absence of randomized controlled trials, these findings may offer clinically important clues about dosing and effectiveness of gabapentin in GAD.

## 2. Case Presentation

### 2.1. Presenting Features

The patient is a 59-year-old Caucasian female. Her psychiatric history is significant for major depressive disorder (MDD) and GAD. She has had four prior inpatient psychiatric hospitalizations for depression and anxiety with suicidality.

Her medical history is pertinent for Hashimoto's thyroiditis with primary hypothyroidism on levothyroxine, primary Sjögren's syndrome (asymptomatic), and autoimmune diabetes with peripheral neuropathy. Social history is pertinent for physical and emotional abuse in childhood. She does not use alcohol, tobacco, or illicit substances. She drinks up to two cups of caffeinated coffee in the morning. Her family history is positive for MDD, GAD, and bipolar disorder.

In the past, the patient failed or did not tolerate treatment with multiple selective serotonin reuptake inhibitors (SSRIs; citalopram up to 100 mg/day, paroxetine 30 mg/day), serotonin-norepinephrine reuptake inhibitors (SNRIs; duloxetine up to 90 mg/day), tricyclic antidepressants (amitriptyline at therapeutic levels and 10 mg/day nortriptyline), mirtazapine 30 mg/day, bupropion 150 mg/day, aripiprazole (discontinued due to concern for excessive gambling), and trazodone. Pregabalin 150 mg/day was helpful for anxiety in the past. She had multiple unsuccessful cognitive behavioral therapy trials. At the age of 52, she was prescribed clonazepam on an “as needed basis” for mild GAD (started on 0.5 mg two times per day (BID) and gradually increased to 1 mg BID).

Three months prior to presentation, benzodiazepines were tapered and the patient's anxiety recurred. Her symptoms included persistent and excessive worry, restlessness, feeling on edge, fatigue, poor concentration, irritability, insomnia, and dysphoric mood, as well as diaphoresis, palpitations, tremulousness, and muscle tension. She was unable to complete activities of daily living. She developed suicidal ideation and required two psychiatric hospitalizations in a one-month span. Ultimately, benzodiazepines were successfully discontinued and she has remained off of these medications throughout 10 months of outpatient follow-up.

During her most recent hospitalization, gabapentin was initiated at a dose of 300 mg three times per day (TID) to manage benzodiazepine withdrawal symptoms. Although she reported some sedation with this dose, she felt “calmer” and her anxiety dissipated. Her dose was titrated to 600 mg TID, and she was discharged from the hospital.

### 2.2. Differential Diagnoses

We considered generalized anxiety disorder, panic disorder, posttraumatic stress disorder, MDD, obsessive compulsive disorder, anxiety due to another medical condition, such as thyroid disease, medication-induced mood, and anxiety disorder from prolonged benzodiazepine withdrawal.

### 2.3. Progression, Treatment, Follow-Up, and Outcome

Apart from gabapentin 600 mg TID, the patient's other discharge medications included doxepin 10 mg at bedtime for sleep, hydroxyzine pamoate 25 mg BID as needed for anxiety, and sertraline 100 mg daily, all of which were discontinued due to side effects or ineffectiveness during subsequent outpatient follow-up. During her outpatient follow-up, anxiety was assessed using a combination of Likert-scale ratings and the generalized anxiety disorder 7-item (GAD-7) patient self-report questionnaire. Four days after discharge, sertraline was increased to 150 mg as per hospital recommendations. She did not tolerate hydroxyzine on as needed basis and stopped using this medication several days after leaving the hospital.

#### 2.3.1. First Gabapentin Discontinuation Attempt

**Figure 1:**
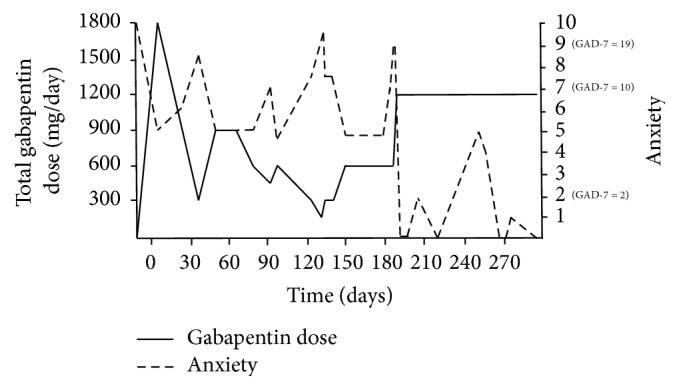
*Relationship between gabapentin dose and anxiety*. Anxiety was assessed on a 0–10 scale; GAD-7 scores are added where available. Day 0 is the day of discharge from the last hospitalization. Gabapentin is expressed in total daily doses. Measurements were not continuous, but for clarity lines connect the values obtained during in-office follow-up appointments or telephone conversations (a total of 27 measurements in 294 days).

On day 18 after discharge, she attempted a self-initiated gabapentin taper due to improved anxiety and perceived “drowsiness” (see Figure 1 for graphical depiction of anxiety-gabapentin dose relationship over time). She decreased the dose to 300 mg TID with no problematic change in her anxiety. On day 30, she requested to stop gabapentin as her anxiety was well controlled. Her outpatient psychiatrist recommended a slow taper, so gabapentin was decreased to 300 mg BID for 1 week. However, her anxiety rapidly returned. Nevertheless, she further decreased gabapentin to 300 mg at bedtime (QHS). At this point, her anxiety became debilitating. On day 48 following discharge, one month after her first self-initiated dose reduction, her gabapentin was again increased to 300 mg TID with immediate and pronounced decrease in anxiety (Figure 1).

#### 2.3.2. Second Gabapentin Discontinuation Attempt

The patient remained on 300 mg TID for approximately one month; however, by day 75, she initiated a second gabapentin self-taper with a reduction to 300 mg BID because she believed that gabapentin was causing her somnolence. She further reduced her dose to 300 mg in the morning and 150 mg QHS on day 77. This change was again accompanied by increased anxiety within days, and 19 days after the last dose reduction, her gabapentin was returned to 300 mg BID with immediate improvement in anxiety (Figure 1).

#### 2.3.3. Third Gabapentin Discontinuation Attempt

On day 118, the patient attempted a third self-initiated gabapentin taper as she believed gabapentin was causing increased fatigue. She first reduced her dose to 300 mg for one week and then further to 150 mg daily for a period of 10 days. However, this was accompanied by another increase in anxiety, and her dose was increased to 300 mg daily. At the same time, sertraline was cross-tapered to amitriptyline to treat chronic migraine. Amitriptyline was titrated to a dose of 50 mg/day, where she achieved a total amitriptyline + nortriptyline concentration of 101 ng/mL [ref.: 80–200 ng/mL]. Doxepin 10 mg was discontinued. Her headache temporarily resolved, but anxiety continued to impact her functioning. During the 2 weeks, while the above-mentioned medication changes were taking place, her anxiety has been increasing but staying in the moderate range on gabapentin 300 mg. As per her request, her dose was increased to 300 mg BID on day 149, followed by a decrease in her anxious symptoms within 24 hours (Figure 1).

#### 2.3.4. Period of Stability

Gabapentin was subsequently continued at a dose of 300 mg BID for approximately 1 month with mild but manageable symptoms present. However, shortly thereafter, the patient had a number of psychosocial stressors that led to increased anxiety (GAD-7 score of 19). Gabapentin was increased to 600 mg BID, and after approximately one month amitriptyline was discontinued as the patient was not convinced of its benefits. Her anxiety decreased to 0/10 within 48 hours of increasing gabapentin, and she remained in remission on gabapentin monotherapy for the next 70 days despite ongoing psychosocial stressors. At the end of this period, she started to develop some depressive symptoms (Patient Health Questionnaire 9 score of 17), though her anxiety remained mild. Venlafaxine was therefore initiated. The patient remains on gabapentin and venlafaxine at the time of manuscript submission.

## 3. Discussion

The potential anxiolytic effect of gabapentin was first observed in animal models [1]. Randomized controlled trials in patients with anxiety disorders found that gabapentin is effective in treating social phobia [2]. Gabapentin was generally not effective in treatment of panic and agoraphobia symptoms. However, a subgroup of more severely ill patients, particularly women, did show some improvement [3].

To our knowledge, there are no controlled studies on gabapentin use in GAD [4]. A compiled study of 18 patients with various anxiety disorders, one of which had GAD, found beneficial effects on anxiety symptoms [5]. Pollack and colleagues published a case report of a patient with GAD who improved on gabapentin 100 mg TID at 3-month follow-up [6]. However, the patient was also receiving 20 mg total daily dose of diazepam [6]. The same author also reported improved anxiety in a patient receiving gabapentin 100 mg BID at 3-month follow-up [6]. However, that patient had alcohol use disorder and had decreased drinking during this period [6]. Despite this lack of efficacy data, our clinical experience indicates that gabapentin is frequently used to treat patients with GAD.

The patient presented here has the most detailed description of gabapentin dose-response on GAD symptoms available in the literature thus far. On average, we contacted the patient every 11 days, for a total of 27 measurements in 294 days of outpatient follow-up. This allows us to determine a detailed dose-efficacy response for this patient. As shown in Figure 1, there was a clear inverse relationship between gabapentin dose and anxiety. Anxiety was rated as low or absent at total daily doses ≥ 900 mg per day. This is particularly encouraging as the patient had multiple failed therapeutic trials of SSRIs, SNRIs, tricyclic antidepressants, bupropion, mirtazapine, aripiprazole, and trazodone targeting anxiety and mood. Compared to these medications, gabapentin has a favorable side effect profile and generally represents lower risk in overdose [7].

The patient was discharged from her last hospitalization on several psychotropic medications including gabapentin, sertraline, doxepin 10 mg QHS, and hydroxyzine 25 mg BID. She quickly discontinued hydroxyzine but continued the other three medications until day 124. During this time, gabapentin was the only medication with repeated dose adjustments, and no other medication changes were temporally correlated with the fluctuating levels of anxiety in this patient. Despite some degree of polypharmacy, the patient's fluctuating anxiety levels were therefore most clearly associated with gabapentin dose changes. Furthermore, starting on day 196, the patient's psychiatric condition was treated with gabapentin 600 mg BID monotherapy for approximately 70 days. She remained in complete remission from anxiety during this period, further supporting the hypothesis that gabapentin at total daily doses ≥ 900 mg/day was effective in treating her GAD symptoms. At the end of this period, the patient developed symptoms consistent with MDD, warranting initiation of venlafaxine; however, she showed no signs of anxious distress, again consistent with gabapentin's efficacy in managing her anxiety symptoms.

In addition to medication concerns, this patient had a particularly high autoimmune disease burden including Hashimoto's thyroiditis, primary Sjögren's syndrome, and autoimmune diabetes. Little is known about the interaction between gabapentin and autoimmune mechanisms. Worsening of myasthenia gravis with gabapentin has been reported, and one case of gabapentin-induced bullous pemphigoid is currently available in the literature [8, 9]. To our knowledge, there have been no controlled studies or case reports indicating that patients with a high autoimmune disease burden should have an altered mood or anxiety response to gabapentin. However, this is a potential consideration that deserves further research. Overall, we believe that no convincing evidence exists at this time to suggest that this patient's favorable response to gabapentin is linked to her autoimmune predisposition or that using gabapentin in such a patient carries an increased risk of adverse events.

We did not measure gabapentin's serum levels in this patient. Firstly, the target serum concentration for treating anxiety with gabapentin is not known and therefore is not clinically useful at this point. Secondly, there was no evidence that the patient falsely reported her gabapentin use, as she was forthcoming with her psychiatrist about her repeated episodes of self-initiated dose reductions and periods of taking the medication as prescribed. Additionally, medication refills were requested and filled at the expected times for the doses that she was taking.

Gabapentin has a complex mechanism of action that only modestly overlaps with other antiepileptics. Its effect is likely mediated partially through inhibition of voltage-gated calcium channels via binding to *α*2*δ*-1 subunits [10]. This affects cellular trafficking of voltage-dependent calcium channels likely causing an overall reduction in calcium currents and potentially impacting neurotransmitter release in currently unknown ways [10]. Binding to this receptor complex has also been postulated to decrease the formation of excitatory neuronal synapses, thus potentially decreasing overall excitatory tone and modulating anxiety [11]. Other potential mechanisms of action that may target anxiety symptoms include modulation of GABA biosynthesis and nonsynaptic GABA neurotransmission [10, 12]. It is notable that although gabapentin does not directly bind to or modulate GABA-A receptors like benzodiazepines, the above mechanisms may indirectly impact GABAergic tone and provide a treatment option not only for anxiety but also for components of both benzodiazepine and alcohol withdrawal [10, 13, 14]. While this patient indeed found gabapentin useful for her benzodiazepine discontinuation, a larger study found that gabapentin was not associated with decreased benzodiazepine use in psychiatric patients [15]. We hypothesize that it was gabapentin's efficacy in treating GAD symptoms that allowed for ongoing benzodiazepine abstinence. Future research is necessary to determine whether patients with GAD who respond positively to gabapentin also have decreased benzodiazepine utilization. This is particularly important given the risks of benzodiazepines in older adults [16].

There has been growing concern about the potential for gabapentin abuse. However, the literature is limited and suggests that gabapentin is predominantly abused by patients with other substance use disorders, notably opioid use disorder [17–21]. The doses used when gabapentin is abused tend to be higher than 3000 mg/day [17]. Our patient showed no signs of abuse or increasing demand for gabapentin and seems to be low risk based on available literature. Nevertheless, it is important for physicians to be aware of the potential for gabapentin abuse when considering prescribing it, particularly for patients with substance use disorders.
